# Architecture of surface tubular element of poxvirus

**DOI:** 10.1128/mbio.03143-25

**Published:** 2026-03-04

**Authors:** Fengxi Yu, Ge Jin, Yixiao Liu, Zhenyu Liu, Jingxuan Yao, Junbo Wang, Daoxin Xie, Zihe Rao, Liming Yan, Yan Zhang, Zixian Sun, Zhiyong Lou

**Affiliations:** 1 MOE Key Laboratory of Protein Science, School of Basic Medical Sciences, Tsinghua University 12442, Beijing, China; 2 Guangzhou National Laboratory, No. 9 XingDaoHuanBei Road, Guangzhou International Bio Island, Guangzhou, Guangdong Province, China; 3 School of Life Sciences, Tsinghua University, Beijing, China; 4 Shanghai Innovation Center for Biopharmaceutical Co., Ltd, Shanghai, China; 5 Department of Applied Biology and Chemical Technology, The Hong Kong Polytechnic University, Hong Kong, China; 6 School of Public Health, Beihua University 74568, Jilin, China; 7 Yanzhao Advanced Biotechnology and Medicine Laboratory, Institute of Tsinghua University, Hebei, China; Case Western Reserve University School of Medicine, Cleveland, Ohio, USA

**Keywords:** surface tubular element, poxvirus, Cryo-EM strucutre

## Abstract

Poxviruses are large enveloped DNA viruses that cause severe human infectious diseases. The mature virion of poxvirus is covered with dense surface tubular elements (STEs), which play a role in assembly progress of mature virions (MVs) and inhibit host cell protein synthesis. However, the composition and assembly of STEs remain unclear. Cryo-electron microscopy (cryo-EM) has proven to be a powerful technique for determining the structure of proteins from complex biological samples. By integrating high-resolution cryo-EM maps with mass spectrometry, we reveal that STEs are helically assembled from two transmembrane proteins, A14 and A17, which bind to phospholipid molecules and form the tubular scaffold along the poxviral membrane. Extensive intermolecular interactions, including A14 dimers and A14-A17 complexes, drive the remarkable structural stability of STEs. Structural analysis further emphasizes the reticulon-like properties of A17, which promote membrane curvature and stabilize the tubular architecture. These results provide novel insights into the STE assembly, morphogenesis, and surface organization of poxviruses, offering valuable information for the development of vaccines and antiviral strategies against poxvirus infections.

**IMPORTANCE:**

Surface tubular elements (STEs) are critical components of poxvirus mature virions and play a role in suppressing host cell protein synthesis. In this study, we isolated and purified STEs from native poxvirus virions and subsequently determined their core composition and high-resolution architecture. We identified that STE is mainly composed of membrane proteins A14 and A17, along with phospholipid molecules. Within the repeat structural unit of STE, A14 proteins form two homodimers within the repeating unit, with A17 monomers flanking either side. Phospholipid molecules are distributed within the A14-A14 and A14-A17 interfaces. Our study not only revealed the molecular structures of A14 and A17 but also further emphasized that the reticulon-like and highly oligomerized characteristics of A17 provide membrane curvature, while the A14-A17-phospholipid network stabilizes the tubular structure. We proposed a hypothetical model that A17 drives changes in viral membrane curvature during maturation. These findings enhance our understanding of poxvirus biology and may guide therapeutic strategies against poxvirus infections.

## INTRODUCTION

The recent emerging Mpox pandemic, with over 100,000 infections, underscores the significant public health threat posed by poxviruses (1). Poxviruses (family *Poxviridae*) are the largest animal viruses, with their genomes encoding more than 200 proteins (2, 3). Within the *Poxviridae* family, the *Orthopoxvirus* genus includes variola virus (also known as smallpox virus, VARV) and Mpox virus (MPXV), posing significant threats to human health (4).

The infection cycle of poxviruses involves a complex, highly coordinated process. Following infection, poxviruses establish viral factories within the host cell, where viral particles are sequentially assembled through distinct stages: the crescent, the immature virion (IV), the mature virion (MV), and the enveloped virion (EV) (5). The crescent, which marks the initiation of virion assembly, originates from the host cell’s endoplasmic reticulum (ER) and is stabilized by transmembrane proteins A14 and A17, as well as the outer lattice protein D13 (6–10). As the crescent grows, it maintains a stable curvature and eventually encapsulates the viral genome to form the non-infectious IV (11–13). During maturation, disulfide bond formation and protein cleavage lead to the appearance of the dense core and lateral bodies within the IV. N-terminal cleavage of A17 disassembles the D13 lattice, forming the asymmetric structure of the mature MV, which gains infectious capability (7, 14–17). The envelope of the MV is enriched with virus-specific lipids and proteins, such as A17 and A27. This process replaces host membrane components and leads to the formation of a distinctive bilayer membrane structure that exhibits high stability, enabling it to withstand external environmental pressures (18, 19). The mechanisms underlying the transition from IVs to MVs, including the dynamic modulation of viral membrane curvature, remain poorly understood. Notably, compared to the IV membrane, the MV membrane incorporates additional proteins that are critical for cell attachment and infection mediation (20, 21).

As key structural proteins, partial topological and structural information for A14, A17, and D13 has been elucidated, with D13 being the most extensively studied (6–8, 22–25). The 63 kDa D13 monomer, which contains two jellyroll domains, assembles into a honeycomb lattice of trimers both *in vivo* and *in vitro* (22). Although lacking a transmembrane domain, D13s function as external scaffolds with strong membrane-remodeling capabilities, attributed to their tight interaction with the N-terminus of A17 (7, 26). Unlike D13, A14, and A17 are co-translationally integrated into the endoplasmic reticulum membrane and are rich in transmembrane domains, remaining anchored to the membrane until MV stage (10). Current research indicates that A14 is a 15 kDa protein that spans the membrane twice, with two transmembrane spans, such that its N- and C-termini face the same side of the membrane, while its central hydrophilic loop is exposed on the opposite side (23). A disulfide bond between the Cys_71_ residues of two A14 monomers forms a stable dimer, enhancing the stability of the viral particle (23). Similarly, the 21 kDa mature A17 protein is also a multi-pass transmembrane protein, but its N-terminus is exposed on the exterior of the virus, while its C-terminus is buried on the other side of the membrane (27). Research has shown that liposomes reconstituted with purified A17 proteins can form 25 nm vesicles at low concentrations and tubules at higher concentrations. Additionally, the overexpression of A17 leads to the transformation of the endoplasmic reticulum (ER) into a highly curved and densely packed network (25). The homo-oligomerization properties of A17, along with its association with the endoplasmic reticulum and its topology, exhibit striking parallels to those of the ER tubule-forming protein DP1/Yop1p, which is known to generate membrane curvature (25, 28). Additionally, A17 serves as an anchor for key adsorption and infection-related proteins, such as A27 and A26, facilitating their attachment to the viral surface through direct or indirect interactions (21, 29).

Imaging techniques such as transmission electron microscopy (TEM), deep tech electron microscopy (DEEM), and atomic force microscopy (AFM) reveal that mature virions (MVs) exhibit a mulberry-like surface densely covered with surface tubular elements (STEs) (18, 19, 30, 31). These STEs are not artificial, as also confirmed by biochemical analyses and described as rod-like structures with a diameter of 20–30 nm, similar in diameter to A17 tubules (18, 19). Initially thought to be composed of a 58 kDa viral protein (p4c or A26), further research indicates that STEs can maintain their integrity under treatment of NP40 and are co-isolable with viral surface proteins, including A17, A26, and A27 (18, 19). Exposure of these STEs to mammalian cells blocks their protein synthesis, as evidenced by decreased polysome formation and increased free ribosomes (32). Moreover, STEs have been proposed to function similarly to the vaccinia cytotoxins reported earlier, which play a crucial role in cell adhesion and membrane fusion (33). Although STEs play critical roles in viral invasion and represent potential immunological targets, their precise composition and assembly mechanism remain poorly understood due to a lack of recent attention in research.

In this study, we successfully obtained high-resolution density maps of the backbone of surface tubular elements (STEs) derived from native poxvirus using cryo-electron microscopy (cryo-EM), achieving a near-atomic resolution of 3.23 Å. By combining mass spectrometry analysis with atomic model building, we identified that the asymmetric unit per helical turn of the helical assembly STEs comprises four A14 proteins, four A17 proteins, and 10 phospholipid molecules. These findings reveal the intricate organization of A14 and A17 within the viral membrane of MV, suggesting that the tightly interconnected A14-A17 network plays a critical role in maintaining the structural integrity of STEs. Based on these results and previous studies, we propose a model in which A17 drives changes in viral membrane curvature. Our findings enhance the understanding of the assembly mechanisms of poxvirus and provide valuable insights for the development of antiviral drugs, such as inhibitors that target viral membrane biogenesis.

## RESULTS

### Sample preparation and component determination

**Fig 1:**
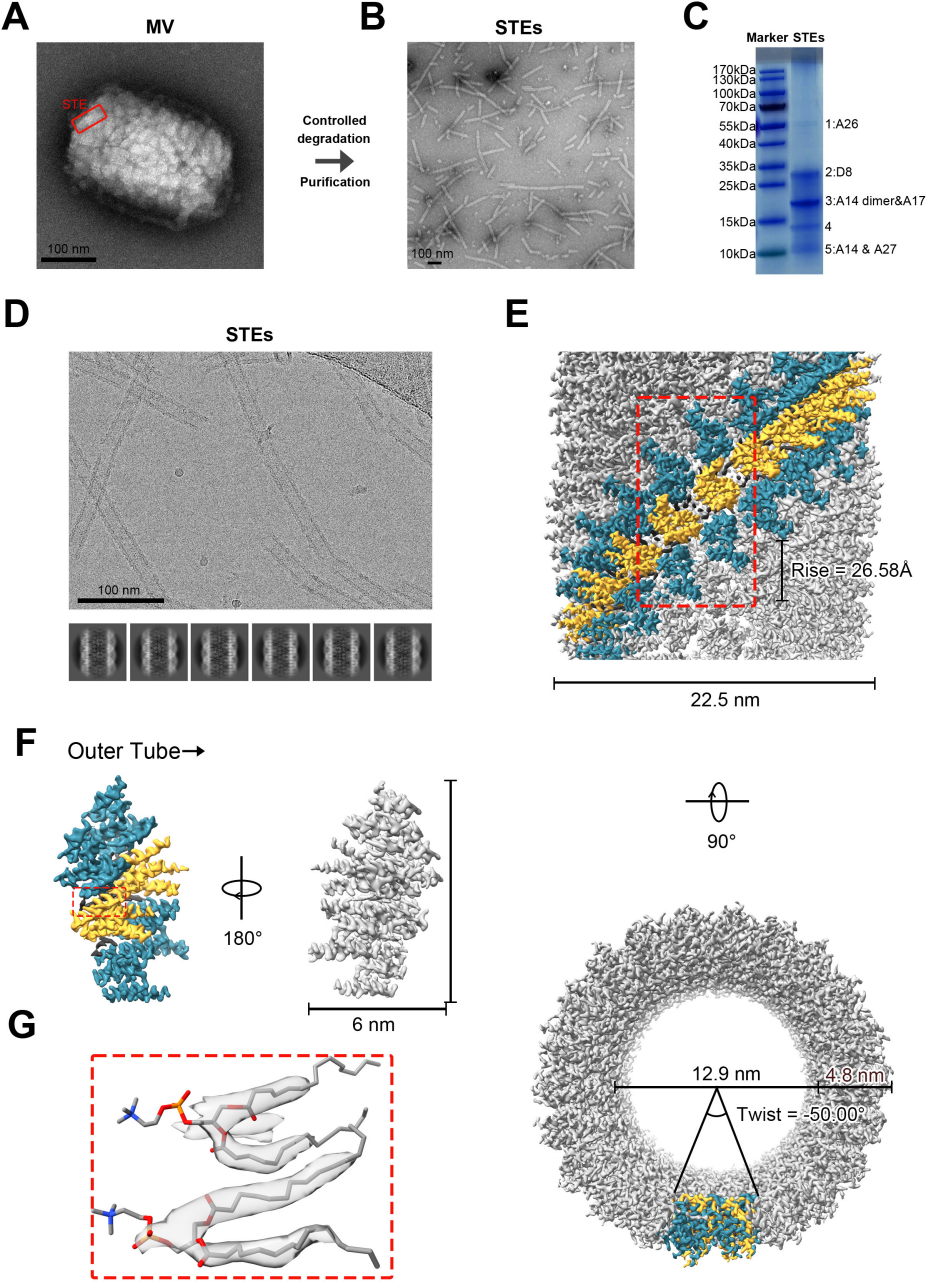
Purification and overall structure of STE. (**A**) A negatively stained electron microscopy (EM) image of MV sample. (**B**) The native mature vaccinia virions have been degraded in a controlled fashion using a NP-40 and β-mercaptoethanol and purified by density gradient centrifugation. Then, purified STEs were observed by negatively stained EM. (**C**) SDS-PAGE analysis of purified STEs. Following the exclusion of contaminated proteins, we identified bands 1, 2, 3, and 5 as corresponding to the STE proteins A26, D8, the A14 dimer and A17, and A14 and A27, respectively. (**D**) A cryo-EM micrograph collected by 300 kV electron microscope showing several STEs. The images below represent 2D classification images with the same diameter. (**E**) The density map of STE determined by helical reconstruction, viewed from different angles. The red rectangle highlights the basic repeating unit of helical reconstruction, as shown in panel F. (**F**) The isolated repeating unit of STE derived from helical reconstruction. A14, A17, and lipids marked in yellow, cyan, and gray, respectively. (**G**) Cryo-EM density (transparent gray) and the atomic model of phospholipid molegrays. Scale bars: 100 nm.

MVs were purified from the non-replicating TianTan vaccinia virus (NTV), which was developed as a replication-deficient vaccine derived from the Tiantan strain of vaccinia virus (TTV) through targeted gene deletion (34). We obtained highly purified MVs by sucrose density gradient centrifugation (Fig. S1A and B; see Materials and Methods for details). Observation of the purified MVs by negative-staining electron microscopy (EM) revealed a rough viral surface due to the presence of dense tubular structures (Fig. 1A and B). Through controlled degradation and sucrose density gradient centrifugation (19, 35, 36), we extracted and purified the structural tubular elements (STEs) from the native MVs (see Materials and Methods for details). Negative-staining EM of the purified STEs showed that they exhibit fibrillar structures, consistent with previous findings (19, 33) (Fig. 1B). Further analysis revealed that the diameter of the purified STEs closely matched that of the analogous structures observed in MVs (Fig. 1A, B and D). SDS-PAGE analysis of the STEs revealed five distinct protein bands (Fig. 1C). Subsequent mass spectrometry identified several principal membrane proteins associated with cell binding and membrane formation (File S1). It should also be noted that, due to the detergent treatment, the mass spectrometry results were heavily contaminated with core proteins and non-membrane-localized proteins, such as A10, while some membrane proteins on the STEs may not remain firmly anchored to the tubular structure. Following the exclusion of contaminated proteins, we identified bands 1, 2, 3, and 5 as corresponding to the STE proteins A26, D8, the A14 dimer and A17, and A14 and A27, respectively (Fig. 1C).

In order to confirm the STE composition accurately, we integrated high-resolution cryo-EM density maps with high-resolution mass spectrometry results, and we identified A14 and A17 as the main protein components of the STEs (Fig. S1C).

### Overall structure of STE

We collected a total of 4,256 sets of 300 kV cryo-EM movies for helical reconstruction. Two-dimensional classification revealed that the tubular structures of STEs were highly consistent and uniform in diameter, exhibiting helical symmetry (Fig. 1D). Through iterative rounds of two-dimensional classification, particle selection, filament tracing, and helical refinement, we obtained the final structure using 477,012 particles, with helical parameters of a twist of −50.00° and a rise of 26.58 Å (Fig. 1E; Fig. S2 and S3; Table S1; Video S1). We determined the cryo-EM structure of NTV STE at the resolution of 3.23 Å (Fourier shell correlation [FSC] = 0.143) using C1 symmetry and helical reconstruction (Fig. 1E; Fig. S2 and S3; Table S1; Video S1).

The overall structure of the STEs exhibits a right-handed helical tubular form, with inner and outer diameters of approximately 129 Å and 225 Å, respectively (Fig. 1E). The dimensions of the basic helical repeating unit are approximately 110 Å × 60 Å × 60 Å (Fig. 1F; Fig. S4). The asymmetric unit within per helical turn of the STEs contains four A14 proteins, four A17 proteins, and ten phospholipid molecules (Fig. 1G; Fig. S5). The A14s interact to form two homodimers that are located centrally within the repeating unit, with individual A17 proteins positioned on each side of the A14 protein. Based on the positions of phospholipids relative to the proteins, we can classify the phospholipids into three types. The first type is located between a single A14 and a single A17. The second is arranged in the groove between one A17 and two A14s. The third is situated in the gap between two A14 dimers. Furthermore, the cryo-electron microscopy structures show that phospholipid molecules are found at the A14-A14 and A14-A17 interfaces (Fig. 1F and G; Fig. S4), and they show a clear sidechain density (Fig. S5).

### Structure of A17 and A14

**Fig 2:**
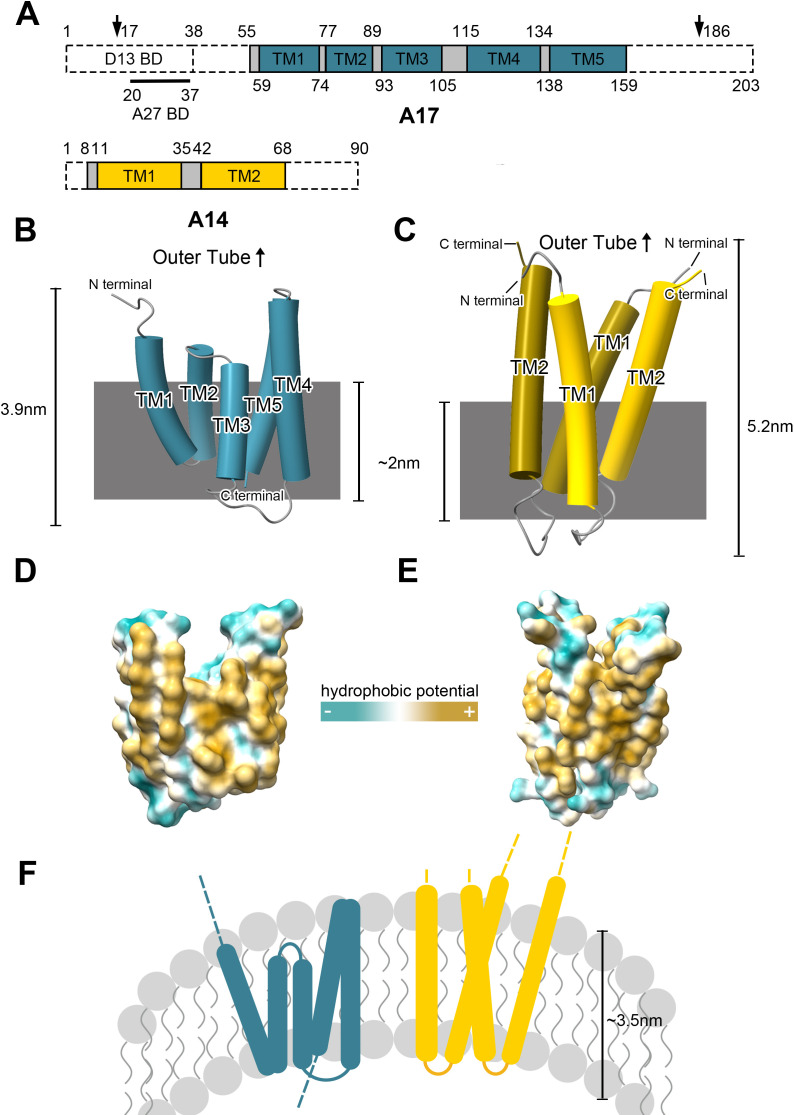
Structure information of base component of STE, A17, and A14. (**A**) Structural layout of the A17 and A14 sequences. The A17 contains a D13 binding domain (BD), an A27 binding domain, and five transmembrane (TM) domains. The A14 contains two TM domains. The dashed box indicates that this portion of the sequence is absent in the density map. The peptide bonds preceding the amino acids indicated by the arrows were previously cleaved by enzymes during the maturation of the poxvirus. (**B and C**) Overall structure of A17 and A14 homodimer. The gray rectangle represents the approximate position of the phospholipid molecule within the density. (**D and E**) Hydrophobic potential of A17 monomer and A14 homodimer. (**F**) Schematic representation of the distribution of the A14 and A17 TM regions within the STE phospholipid bilayer.

In the cryo-EM density, residues 55–159 of A17 and residues 8–68 of A14 can be well traced, while the C- and N-terminal regions of both A17 and A14 are missing in the density (Fig. 1G and 2A). Both A17 and A14 are primarily composed of five and two transmembrane (TM) α-helices, respectively. The overall structure of A17 is reticulon-like. The first two TMs form a V shape structure, with only two residues (S75 and P76) at the turn. TM3 and TM4 contain nine residues between the two helices, forming a U-shaped structure. In addition, TM3-5 forms a triple helix, and extensive hydrophobic interactions between them stabilize A17 within the lipid bilayer (Fig. 2B). This structural characterization is highly similar to the reticulon homology domain (RHD) of ER tubule-forming proteins (Fig. S6). The wedge-shaped insertion allows the protein to occupy more space on the outer leaflet of the lipid bilayer, thereby contributing to the generation of membrane curvature (25, 28). Previous studies have shown that the processed N-terminus of A17 during the MV stage features several outward-facing residues that facilitate binding with A27 (37). However, the density corresponding to the N-terminus of A17 could not be observed in our cryo-EM reconstruction. A14 has longer TMs with bends between them. Two A14 molecules closely intertwine to form a reticulon structure, instead of presenting a wedge-shaped hairpin like the A17 (Fig. 2C).

The turn regions of A17 and A14 are rich in hydrophilic residues, whereas their transmembrane regions are predominantly hydrophobic (Fig. 2D and E). This suggests that the regions near the inner or outer sides of the tubular structure tend to protrude outward from the phospholipid bilayer. Additionally, cryo-EM data reveal the distribution of phospholipids on the inner side of STE, with their hydrophilic heads oriented outward. The lengths of the A17 protomer and A14 dimers are 3.9 nm and 5.2 nm, respectively (Fig. 2B and C), exceeding the assumed bilayer thickness of 3.5 nm (28). The solvent-exposed N- and C-terminal of A14 and A17, which protrude the membrane, facilitate interactions with other viral proteins (6, 8, 9, 23).

### Protein subunit interactions reveal assembly and curvature generation of STE

**Fig 3:**
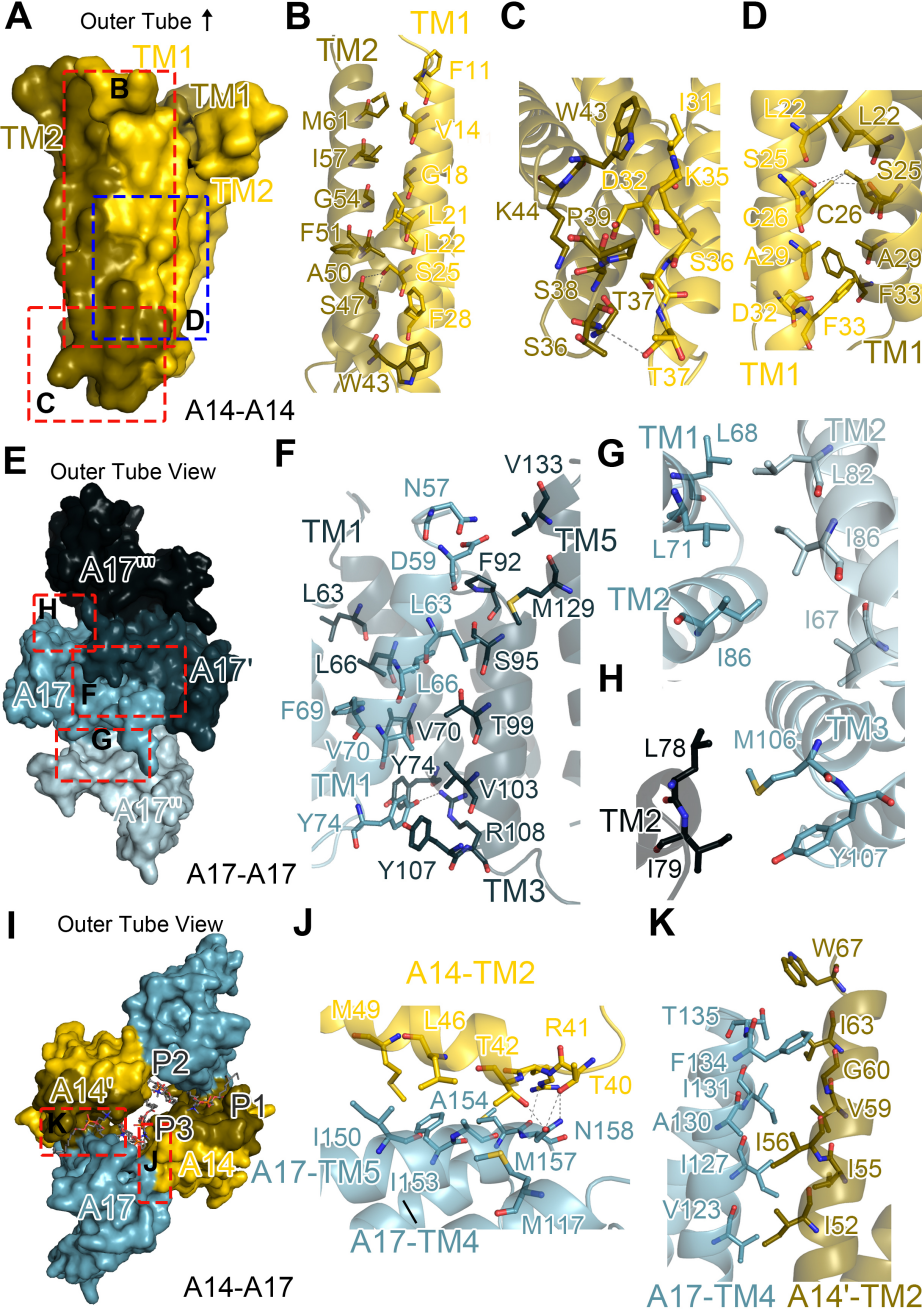
Contacts between subunits in STE. (**A**) The overall structure of analyzed A14 dimer is depicted, with subunit contacts highlighted in red or blue. (**B**) A zoomed-in view illustrates the contacts between A14-TM1 and A14-TM2, corresponding to panel **A**. (**C**) Similarly, this panel shows the contacts between A14-loop/TM1 and A14-loop/TM2, also corresponding to panel **A**. (**D**) A zoomed-in view of contacts between A14-TM1 and A14-TM1, corresponding to panel **A**. (**E**) The overall structure of analyzed A17s. For clarity, distinct colors and alphabetical designations are used to identify various monomers. (**F–H**) Interactions between A17-A17', A17-A17'', and A17-A17''', respectively. (**I**) The overall structure of analyzed A14s, A17s, and phospholipids. Different colors and letters represent different monomers, related to panels **A **and **E**. (**J and K**) Interactions between A17 and two A14s, as well as another A17 from the same interactions within the repeating unit, specifically A17-A14' and A17-A14''. Dashed lines indicate potential hydrogen bonds between the atoms.

To better understand assembly of STE, we analyzed the subunit interactions within the STEs, including those between A14-A14, A17-A17, and A14-A17 (Fig. 3). The intramolecular interactions of the A14 homodimer are the strongest, playing a critical role in stabilizing the STE structure. The interaction area between A14 protomers in one A14 homodimer is 1,579 Å², compared to the surface area of an A14 protomer (5,460 Å²). Nearly half of the residues within the TM1-TM2 interface mediate interprotomer interactions in the A14 dimer (Fig. 3A through C; Table S2). More than half of the residues involved in these interactions are hydrophobic, suggesting that the stability of the A14 dimeric structure primarily depends on the hydrophobic interaction. Key residues involved in the hydrophobic interaction are F11, V14, L21, S25, F28, A29, I31, and D32 from TM1, and W43, K44, S47, A50, F51, I57, and M61 from TM2 (Fig. 3B and C; Table S2). The loop regions of the two A14s are in close proximity and rely mainly on hydrophilic residues for contacts, including K35, S36, T37, S38, and P39 (Fig. 3C; Table S2). Two A14-TM1 helices face each other, forming an inclination of approximately 20 degrees. Residues L22, S25, C26, A29, D32, and F33 in A14-TM1 make contact with each other. Furthermore, a large number of hydrogen bonds are observed between A14 homodimers, including S25-S47 (TM1-TM2), T37-T37 (loop-loop), and an S25-C26/C26-C26 hydrogen bond network (TM1-TM1) (Fig. 3D; Table S2). These hydrogen bonds provide additional stabilization for the A14-A14 interaction beyond hydrophobic interactions. Notably, due to the considerable distance between the C26 residues (3.47 Å) and the possible addition of a reducing agent during sample preparation, no disulfide bond was detected. Additionally, C71, which has been reported to be necessary and sufficient for disulfide bond formation between A14s and A14 dimerization (23), was also not observed in our cryo-EM map. Furthermore, no interaction was detected between the two TM2 helices of A14 due to the distance between them.

In contrast to A14, A17 exhibits a homo-oligomeric morphology (Fig. 3E), consistent with the curvature generation mechanism by Yop1p (28). The homo-oligomerization of the reticulon-like A17 protein promotes membrane curvature, facilitating the formation of tubular structures (25, 28). Within the helical STE, three distinct interaction interfaces exist among A17 molecules. To clearly visualize and analyze these interactions, we designate the four adjacent A17 molecules as A17-A17''' and render each in a distinct color for enhanced structural discrimination (Fig. 3E). The strongest interaction occurs between A17 and A17', forming a dimer with an interface area of 1,042 Å². In this configuration, the N-termini of A and B face each other, with TM1 of one A17 interacting with TM1, TM3, and TM5 of the other (Fig. 3F). The residues involved in the A17 dimer interaction are predominantly hydrophobic. Specifically, residues L66, F69, V70, and Y74 comprise the TM1-TM1 interface. Residues V70, Y74, S95, T99, V103, Y107, and R108 participate in TM1-TM3 interactions, while D59, L63, M129, and V133 mediate TM1-TM5 interactions (Fig. 3F; Table S3). Two hydrogen bonds, Y74-Y74 and Y74-R108, further stabilize the A17 dimer (Fig. 3F; Table S3). The A17-A17'' interaction involves fewer residues. The TM1-TM2 contact is prominent, marked by hydrophobic interactions and an interface area of 337 Å², contributed by residues I67, L68, L71, L82, and I86 (Fig. 3G; Table S3). The A17-A17''' interaction is the weakest, consisting of two pairs of hydrophobic residues from TM3 (M106 and Y107) and TM2 (L78 and I79), with an interface area of 97.3 Å² (Fig. 3H; Table S3). In conclusion, the interactions between A17 molecules are predominantly hydrophobic, with hydrogen bonds playing a minor role. These findings indicate that A17s utilize four dimer interfaces, primarily involving TM1, to achieve homo-oligomerization and drive the assembly of the STE structure.

The oligomers of different homologous units are connected via the A14-A17 interface. Similarly, for unambiguous visualization, the adjacent two A14 molecules were designated A14 and A14', while the three associated phosphate groups were assigned the labels P1, P2, and P3 (Fig. 3I). Two distinct interaction forms exist between A14 and A17: A14-A17 and A14′-A17 (Fig. 3I). For the A14-A17 interaction between F and E, A14-TM2 predominantly interacts with A17-TM5, covering an interface area of 325 Å² (Fig. 3J). The residues involved in this interaction are T40, R41, T42, L46, and M49 from A14, and M117, I150, I153, A154, M157, and N158 from A17 (Fig. 3J; Table S4). Multiple hydrogen bonds, including T40-N158, R41-M157, and T42-N158, stabilize the interaction between A14-TM2 and A17-TM5. Additionally, each A14 interacts with the TM4 of an adjacent A17 protein through its TM2 (A14 and A14' in Fig. 3I), forming an interface area of 363 Å² (Fig. 3K). This interaction is mediated entirely by hydrophobic forces, involving residues I52, I55, I56, V59, G60, I63, and W67 from A14′-TM2, and V123, I127, A130, I131, F134, and T135 from A17-TM4 (Fig. 3K; Table S4).

Previous studies have demonstrated that A17 alone is sufficient to form tubular structures with liposomes (25). Our structural analysis reveals that TM1, TM3, and TM5 of A17 provide homodimerization interfaces. While this arrangement contributes to stability, the wedge-shaped structure of the polymer also induces curvature, facilitating the formation of the tubular structure. On the other hand, the strong interactions of A14 dimers, along with the numerous interactions between heterologous proteins, provide axial stability for the tubule.

### Structure of the phospholipid molecules within the STEs

**Fig 4:**
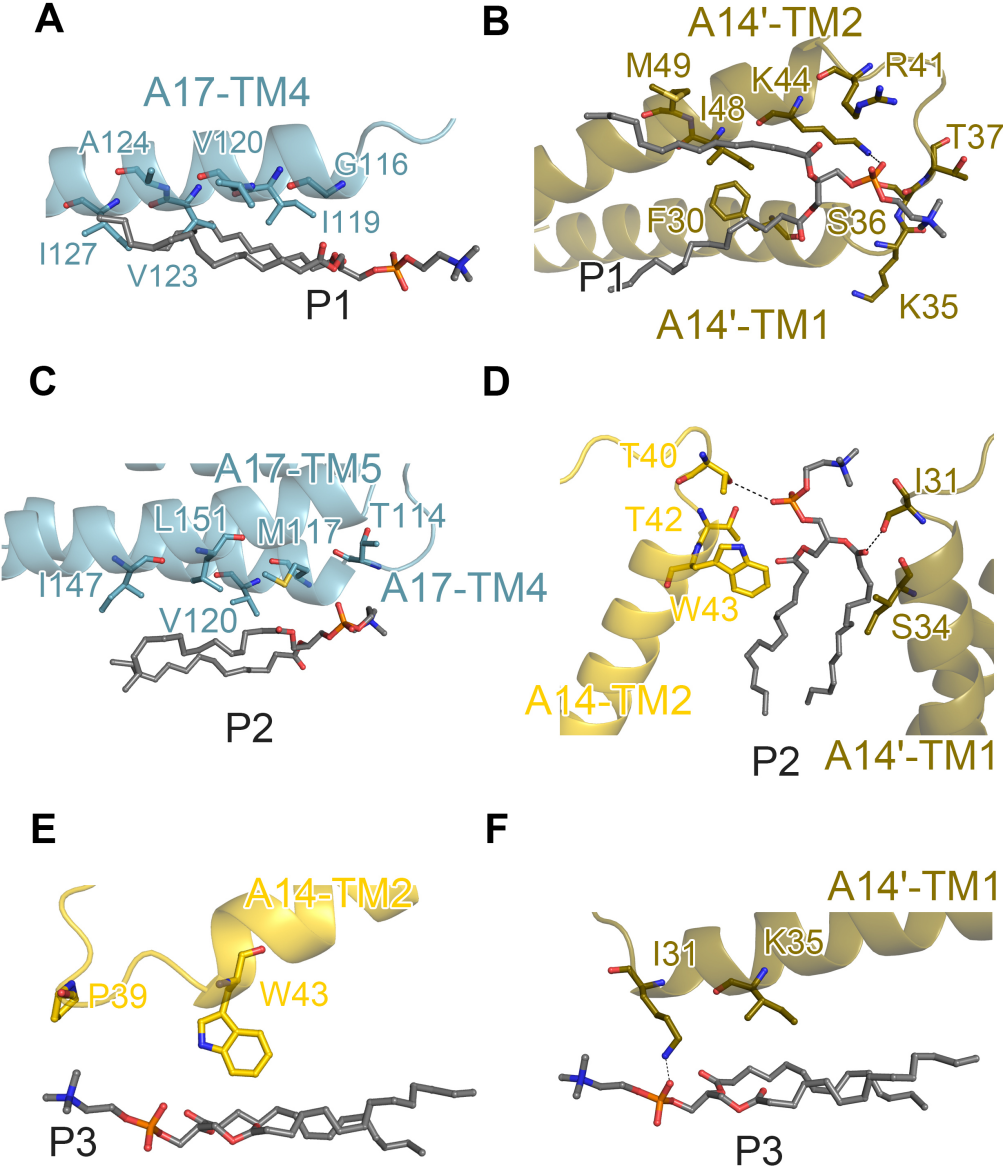
Interaction of proteins and phospholipid molecules in STE, related to Fig. 3H. (**A–F**) Analysis of interactions between proteins and phospholipid molecules in Fig. 3I, which represents the specific interactions between P1-A17, P1-A14', P2-A17, P2-A14 and P2-A14', P3-A14, and P3-A14'. The dashed lines indicate potential hydrogen bonds between the atoms.

In STEs, we identified phospholipid molecules distributed at the interface between A14 and A17, which can be categorized into three distinct classes (Fig. S4 and S5). Notably, the ER tubular protein Yop1p requires only a small number of lipid molecules to form tubules, without distinguishing between lipid types (38). For clarity, these phospholipids were designated as P1, P2, and P3 (Fig. 3H). P1 exhibits two hydrophobic tails that span the A14–A17 interface, forming interactions with both components. Specifically, A17 establishes interactions with the tails of P1 via residues G116, I119, V120, V123, A124, and I127 in TM4 (Fig. 4A). A14' interacts with P1 through residues F30, K44, I48, and M49 in the transmembrane regions, while polar residues K35, S36, T37, and R41 in the loop region, along with K44 in TM2, interact with the phospholipid head group (Fig. 4B).

P2 is embedded within the hydrophobic cavity formed by A17 and its two adjacent A14 molecules. In this interaction, residues T114 and M117 of A17 contact the phospholipid head, while V120, I147, and L151 interact with the hydrophobic tails of P2 (Fig. 4C and D). Additionally, residues T40, T42, and W43 in A14, along with S34 in TM2 of A14', make contact with P2. Notably, I31 and T40 form hydrogen bonds with the tail and head of P2, respectively, while the remaining residues contribute to stabilizing the hydrophobic tail.

The third class of phospholipid (P3) is located between two A14 molecules that do not form a dimer. In this configuration, residues I31 and P39 interact with the hydrophilic head of P3, while K35 and W43 contact the hydrophobic tails (Fig. 4E and F). The interactions between A14, A17, and the phospholipids are predominantly hydrophobic, relying on close contacts between the transmembrane regions and the hydrophobic tails of the phospholipids. These interactions play a critical role in maintaining the structural integrity and transverse solidity of the tubular structure.

## DISCUSSION

Poxviruses, with their large size, complex composition, and pleomorphic nature, pose significant challenges to understanding their molecular architecture, impeding our understanding of the mechanisms underlying their assembly. Surface tubular elements (STEs), first identified in the 1970s (18), have long remained enigmatic in terms of their structure and function. In this study, we elucidated the core components, spatial arrangements, and molecular structure of STEs through the integration of high-resolution cryo-EM density maps with high-resolution mass spectrometry. Our findings revealed that STEs are composed of A14, A17, and phospholipid molecules, organized in a highly helical symmetric arrangement. The structure of STEs shows that the fundamental repeating unit of the STE exhibits a stoichiometry of 4:4:10 for A14, A17, and phospholipid. Structural analysis revealed that A14 proteins form two homodimers within the repeating unit, with A17 monomers flanking either side. Ten phospholipid molecules are distributed within the A14-A14 and A14-A17 interfaces. The stability of the STE architecture is underpinned by strong interactions at the A14-A14, A17-A17, and A14-A17 interfaces.

A14 and A17, two key proteins in the original viral membrane structure (the crescent), continue to serve as major components of the external scaffold during viral maturation (5, 6, 8). As membrane protein scaffolds for the crescent and immature virion, mutations in either A14 or A17 disrupt normal viral particle production (10, 39). Our study elucidates the molecular structures of both A14 and A17, while underscoring the crucial role of strong interactions within A14 dimers and at the A14-A17 interface in maintaining the structural stability of the viral membrane and the STE. These interactions likely constitute the core assembly interface.

Previous studies have indicated that A27 (residues 85–110) can specifically bind to two active binding sites on A17 (residues 20–29 and residues 32–36) and likely exists in a trimeric conformation (37, 40). By integrating the structure in STE with previous research, A27 dimers were found to bind A17 dimers (8, 41). We inferred potential A27-STE binding modes by predicting A27 trimer-A17 dimer model generated through AlphaFold 3 (42). Notably, over 50% and 60% of the A27 and A17 sequences, respectively, exhibit pLDDT scores exceeding 70 (Fig. S7B and C). The predicted full-length A27 protein intertwines in a helical arrangement (Fig. S7B and E). The TMs of the predicted A17 molecules align well with the experimentally resolved A17 structure, also confirming the reliability of the prediction (Fig. S7C, D and F). The model suggests that residues 18–38 of two A17 molecules interact with residues 84–110 of A27 (Fig. S7C and G), which is consistent with previous reports (37). Given the dense distribution of STEs on the surface of MVs, this A17-A27 binding configuration is well-suited to facilitate viral adsorption and fusion to host cells (19, 29).

Although three-dimensional structures of ER tubule-forming proteins remain unresolved experimentally, predictive modeling and functional studies have identified key structural determinants. These include (i) transmembrane (TM) hairpins, (ii) amphipathic helices (APH) at terminal termini, (iii) cytoplasmic loops (cytL), and (iv) C-terminal helices (CTH) (28, 43, 44). These elements collectively promote oligomerization, stabilizing membrane curvature, with the TM hairpins specifically driving curvature generation. Notably, the spatial arrangement of these components can vary significantly, as illustrated by the distinct architectures of hairpins and cytLs observed in RTN4 compared to Yop1p (28). Given that our experimental structure of A17 is incomplete due to the absence of the C- and N-terminal regions, we utilized the full-length structure predicted by AlphaFold 3 for comparative analysis (Fig. S6B). This approach was validated by the high consistency between the predicted structure and our experimental data (Fig. S6B and S7D). Structural analysis revealed that A17 shares several similarities with known ER tubule-forming proteins, including the presence of multiple TMs, nAPH, and cytL that drive oligomerization. A17 also possesses TM hairpins that contribute to membrane curvature. However, A17 exhibits unique structural features: it lacks the surface-exposed cAPH and CTH helices observed in other ER tubule-forming proteins. Instead, A17 contains an additional TM5 domain that traverses the lipid bilayer, positioning its C-terminus within the membrane interior. This distinctive structural arrangement likely enables A17 to perform unique functional roles during virion maturation and assembly.

**Fig 5:**
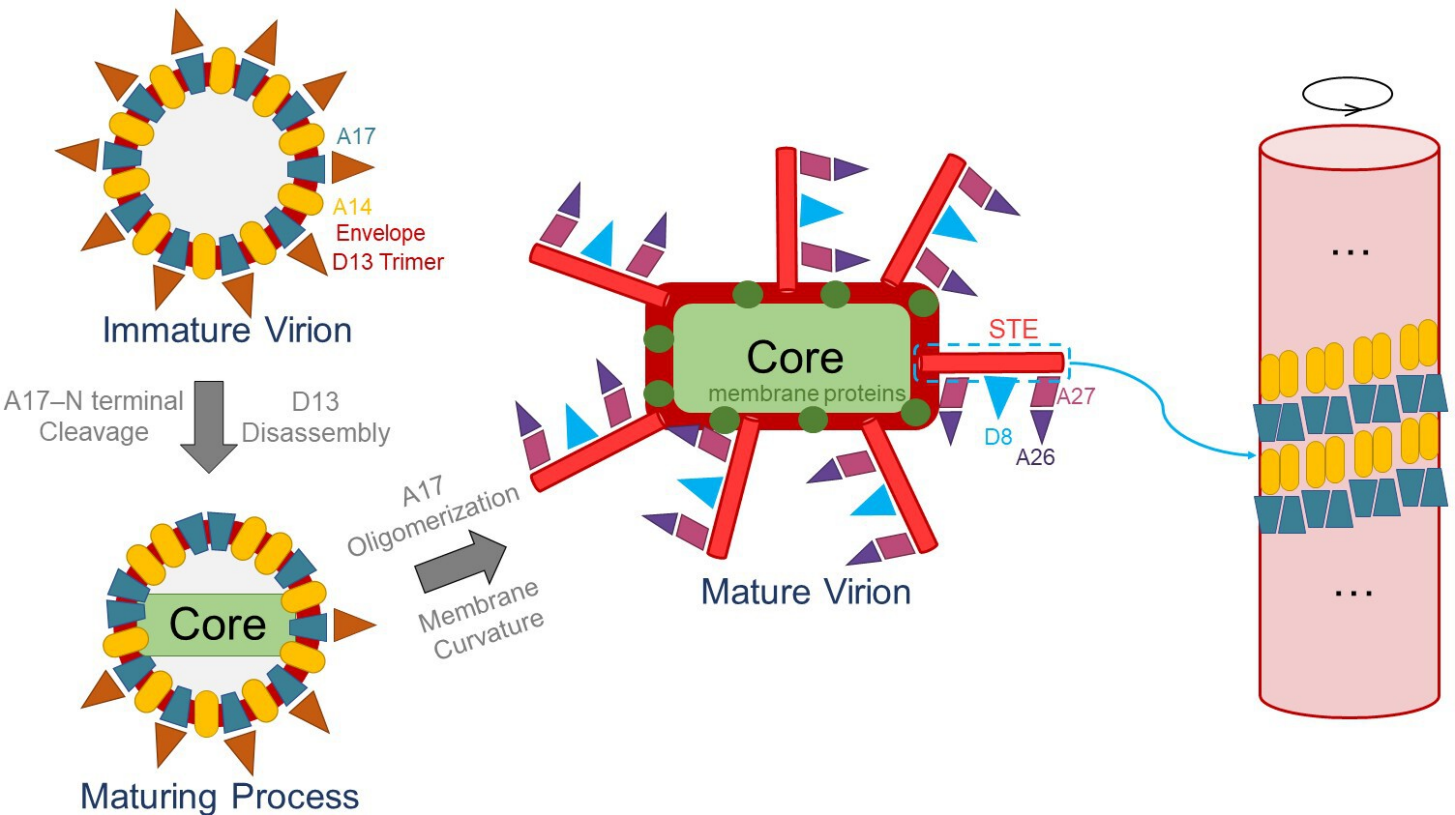
Hypothetical model of STE assembly and A17-mediated membrane curvature in poxvirus. In the IV stage, the N-terminal region of A17 interacts with the D13 lattice, which is densely packed on the IV membrane. At this stage, A14 and A17 are alternately arranged, with A17 existing predominantly in a non-oligomeric state. The disassembly of D13s causes A17s to rearrange with other membrane components, causing A17s to regain oligomerization. This reorganization facilitates the formation of a tubular scaffold (STEs) on the viral surface, driving the morphological transition of viral membrane from the D13’s spherical to the A14-A17’s tubular curvature. STEs serve as anchors for multiple membrane proteins, including A27, A26, and D8.

During the IV stage of VACV, D13s attach to the spherical IV surface as scaffold proteins. Later, as the maturation of virion, the N-terminus of A17 is cleaved. This is followed by the disappearance of the additional curvature provided by D13, and the virion surface transforms from spherical D13s to a dense STEs. The mechanisms governing the structural transition of the virion surface during this process remain poorly understood. As previously indicated, the oligomerization of reticular proteins is an important factor in generating and stabilizing membrane curvature (28, 43). Based on the cryo-EM structure of STE and previous researches (5, 7, 8, 18, 20–23, 40, 45), we propose a hypothetical model (Fig. 5): each D13 trimeric channel accommodates six residues of the A17 N-terminal peptide, and in this D13-bound state, A17 likely exists in a non-oligomeric form dispersed at the IV membrane. We speculate that the disassembly of the D13 scaffold could cause A17 to rearrange with other membrane components, potentially allowing it to regain its ability to oligomerize. This reorganization might then facilitate the formation of a tubular scaffold (STEs) on the viral surface, driving the morphological transition of the viral membrane from the spherical curvature imposed by D13 to the tubular curvature of the A14-A17 complex. In this scenario, the dissociation of D13 from A17 may serve as a critical triggering event, freeing A17 to rearrange and initiate the assembly of the STE scaffold, akin to the start of a multistep cascade. We further postulate that, functioning as anchors within these STEs, A17 could then recruit other proteins, such as A27 and A26, to associate with the MV membrane. During this period, the binding proteins D8 also bind to STE. It is plausible that A17 promotes membrane curvature via its intrinsic properties or through cooperation with other viral/host proteins following D13 release. Although the STEs could potentially act as signaling platforms, their precise functional role in poxvirus replication remains to be fully defined (46). Future studies are required to dissect the molecular details of the A17-D13 interaction, the structural dynamics involved, and their spatiotemporal regulation, which will deepen our mechanistic understanding of this critical morphological transition. It is important to note that this is a hypothetical model, which necessitates further validation under conditions that more closely recapitulate physiological environments. Of note, A14, A17, and A27 are highly conserved among poxviruses (Fig. S8 through S10). This proposed model could be a general model for all poxviruses, including the Mpox virus. These results will be helpful to further understand the underlying biology of poxvirus and to develop therapeutics against poxvirus infections, making it a key target for antiviral drugs and neutralizing antibody (40, 47).

In summary, we employed high-resolution cryo-EM and mass spectrometry to characterize the core components and molecular architecture of STEs purified from native poxvirus particles and to elucidate the assembly mechanism of STEs. Moreover, integrating our experimental findings with prior evidence, we propose a model whereby A17 facilitates membrane curvature induction during poxvirus maturation. While biosafety level constraints prevented us from conducting biochemical and cellular assays to track the dynamic changes of A14 and A17 during poxvirus maturation, we plan to collaborate with researchers in high biosafety level facilities for further functional studies. These findings advance our understanding of poxvirus biology and guide the development of therapeutic strategies against poxvirus infections.

## MATERIALS AND METHODS

### Virus purification

The culture and purification of the non-replicating TianTan vaccinia virus (NTV) were conducted by Beijing Institute of Biological Products of Sinopharm Group. The purification procedure followed previously established protocols (19). Infected chicken embryo cells were resuspended in phosphate-buffered saline (PBS) buffer containing 1% NP-40 and subjected to sonication for cell disruption. Following sonication, 0.06% trypsin was added to the mixture, and the sample was incubated at 37°C for 30 min to digest proteins. The mixture was then centrifuged to pellet cellular debris. The supernatant was subjected to two successive centrifugations at 13,500 rpm for 80 min using 36% and 40% sucrose density gradients. The final pellet, containing purified virions, was resuspended in PBS buffer for further use.

### Purification and isolation of STEs

The isolation of STEs was adapted from previously described protocols (19, 33). Virion samples were centrifuged using an SW41 rotor (Beckman Coulter, USA) 20,000 × *g* for 90 min. The resulting pellet was resuspended in 10 mM Tris-HCl buffer (pH 8.0, referred to as TB Buffer) and treated with NP-40 and β-mercaptoethanol to achieve final concentrations of 0.1% and 0.5%, respectively. The mixture was incubated at 37°C for 30 min, followed by the addition of 100 μL of the mixture onto a 0.45 mL 40% sucrose cushion. This was centrifuged for 20 min using an MLS50 rotor (Beckman Coulter, USA) at 105,000 × *g*. After centrifugation, the TB overlay (including 100 μL of the sucrose cushion) was diluted with TB Buffer to a final volume of 4 mL and centrifuged again using an MLS50 rotor at 105,000 × *g* for 18 h. The sediment containing STEs was resuspended in 20 μL of TB Buffer. For further purification, the suspension was subjected to centrifugation at 15,000 × *g* for 30 min through a 0–50% sucrose gradient. The purified STE suspension was collected from the top 2–3 mL of the gradient.

### LC-MS/MS analysis

The digested protein solution was analyzed using the Thermo Scientific Vanquish Neo UHPLC system. A 60-minute gradient elution was performed at a flow rate of 0.300 μL/min. The eluent was directly interfaced with a Thermo Orbitrap Ascend Tribrid mass spectrometer. The analytical column consisted of a fused quartz capillary (100 μm internal diameter, 40 cm length) packed with C-18 resin (120 Å, 1.9 μm, Dr. Maisch, Ammerbuch, Germany). Mobile phase A was 0.1% formic acid, while mobile phase B was 80% acetonitrile with 0.1% formic acid.

The Orbitrap Ascend Tribrid mass spectrometer was operated in data-dependent acquisition mode using Xcalibur 4.6 software. A full-scan mass spectrometry mode was applied with a mass range of 350–1,600 m/z and a resolution of 240,000. MS/MS scans were performed in the Orbitrap with optimized efficiency.

The Vaccinia Virus database was obtained from UniProt using Proteome Discoverer (Version PD 3.0, Thermo Fisher Scientific, USA). MS/MS spectra from each LC-MS/MS run were acquired and analyzed. The search parameters were set as follows: trypsin digestion with full specificity, allowing up to two missed cleavages. Variable modifications included oxidation (M), acetyl (N-terminal), met-loss (M, protein N-terminal), and met-loss + acetyl (M, protein N-terminal), while carbamidomethyl (C) was applied as a fixed modification. Mass tolerance settings were stringent: precursor ions were analyzed with a 20 ppm tolerance in all Orbitrap-captured mass spectra, and fragment ions were measured with a 0.02 Da tolerance in all MS2 spectra. The false discovery rate (FDR) for peptides was calculated using the Percolator tool within Proteome Discoverer (PD), based on peptide-spectrum matches (PSMs) generated from searches against a reverse decoy database. Peptides uniquely matching a specific protein group were designated as distinct. A stringent FDR threshold of 0.01 was applied to validate protein identifications.

### Negative staining and electron microscopy

Glow-discharged 200-mesh carbon-coated copper grids (Quantifoil, Germany) were prepared by adsorbing 5 μL of the sample onto the grid surface for 2 min. The grids were then rinsed three times with a 2% uranyl acetate solution for 10 s each and subsequently blot-dried. Imaging was performed using a 120 kV FEI Tecnai Spirit electron microscope (Thermo Fisher Scientific, USA).

### Cryo-EM sample preparation and data collection

An aliquot of 5 μL of sample was loaded onto a glow-discharged, carbon-coated copper grid (Cu 1.2/1.3+C, 300 mesh; Quantifoil, Germany). The grid was then blotted for 3.5 s with a blot force of 0 in 100% relative humidity and plunge-frozen in liquid ethane using a Vitrobot Mark IV (Thermo Fisher Scientific, USA).

To obtain a high-resolution structure of the STE, cryo-EM data were acquired using a 300 kV FEI Titan Krios G3 electron microscope (Thermo Fisher Scientific, USA) equipped with a K3 direct detection camera. Micrographs were collected at a magnification of 29,000×, yielding a pixel size of 0.82 Å. In total, 4,256 movie series were recorded, each with an accumulated electron dose of 60 electrons per Å² per second. Each movie was fractionated into a stack of 32 frames, with a defocus range of −2.5 to −1.0 μm and a spherical aberration of 2.7 mm. Statistics for data collection and refinement are summarized in Table S1.

### Cryo-EM image processing

4,256 sets of movies were processed using MotionCor2 (v1.2.4) (48). After patching CTF estimation in cryoSPARC4 (49), we selected the first 1,000 micrographs and applied template-free filament tracer program, followed by one round of 2D classification to obtain 3,019 particles for Topaz training (50). After training, we first extracted particles in 224 pixels and applied multiple rounds of 2D classifications for getting 477,012 particles. After subset selection, initial symmetry search, and helix refinement with symmetry search, the initial model reconstruction of 244,153 particles with parameters of twist −59.2° and rise 27.84 Å was generated. Next, we re-extract all particles with 448 pixels and apply multiple rounds of helix refinement with finer symmetry search range, local CTF refinement, and helix NU-refine. Finally, we reconstructed the 3D STE helical structure with C1 symmetry from 477,012 particles at a global resolution of 3.23 Å, based on the gold-standard Fourier shell correlation criterion of FSC = 0.143, with final twist −50.00° and rise 26.58 Å. The workflow is shown in Fig. S2.

### Model building and refinement

The atomic model was manually built in WinCoot (v0.9.8.92), and further iterations were refined in Phenix (v1.20.1-4487) using real-space refinement (51, 52). The geometry and statistics are given in Table S1. Molecular representations were generated and analyzed with PyMOL (the PyMOL Molecular Graphics System, Version 3.1 Schrödinger, LLC) and UCSF ChimeraX (v1.6.1) (53).

### AlphaFold 3 structure prediction

Structural prediction was performed with AlphaFold 3 server (https://golgi.sandbox.google.com/). The protein sequences used in prediction correspond to the following Uniprot accession numbers: A14: Q76ZQ3; A17: P68593; A27: P11258; Yop1p: Q12402; REEP1: Q9H902; REEP5: Q00765; RTN4: Q9NQC3. Comparisons and illustrations were prepared using the program PyMOL.

## Data Availability

The cryo-EM density map and the structure of STE were deposited into the Electron Microscopy Data Bank (EMDB) and Protein Data Bank (PDB) under accession numbers EMD-63964 and 9U9H, respectively. All other data supporting the findings of this study are available from the corresponding authors upon request.

## SUPPLEMENTAL MATERIAL

File S1 mbio.03143-25-s0001.xlsx Raw data from the mass spectrometry analysis of the protein composition of purified STE.

Supplemental material mbio.03143-25-s0002.pdf Fig. S1 to S10; Tables S1 to S4.

Video S1 mbio.03143-25-s0003.mp4 STE helical reconstruction and demonstration of asymmetric structural units.

ASM does not own the copyrights to Supplemental Material that may be linked to, or accessed through, an article. The authors have granted ASM a non-exclusive, world-wide license to publish the Supplemental Material files. Please contact the corresponding author directly for reuse.
