# Supplementary material for: Architecture of surface tubular element of poxvirus
Source: mBio. 2026 Mar 4;17(4):e03143-25. doi: 10.1128/mbio.03143-25 (PMC13059796; doi:10.1128/mbio.03143-25)
Supplement: Supplemental material — Fig. S1 to S10; Tables S1 to S4. [file mbio.03143-25-s0002.pdf]

Supplementary Information for:

Architecture of Surface Tubular Element of Poxvirus

Fengxi Yu, Ge Jin, Yixiao Liu, Zhenyu Liu, Jingxuan Yao, Junbo Wang, Zihe Rao, Daoxin Xie, Liming Yan, Yan Zhang, Zixian Sun, Zhiyong Lou

Contents **Page**

Supplementary Figures 3

Supplementary Figure 1........ 4

Supplementary Figure 2........ 7

Supplementary Figure 3........ 8

Supplementary Figure 4........ 9

Supplementary Figure 5........ 10

Supplementary Figure 6........ 11

Supplementary Figure 7........ 14

Supplementary Figure 8........ 16

Supplementary Figure 9........ 17

Supplementary Figure 10........ 18

Supplementary Tables 19

Supplementary Table 1. Cryo-EM data collection, refinement and validation statistics 19

Supplementary Table 2. Interactions between A14 dimer........ 20

Supplementary Table 3. Interactions between A17-A17. 21

Supplementary Table 4. Interactions between A14-A17. 22

Reference.. 23

Supplementary Figures

Supplementary Figure 1

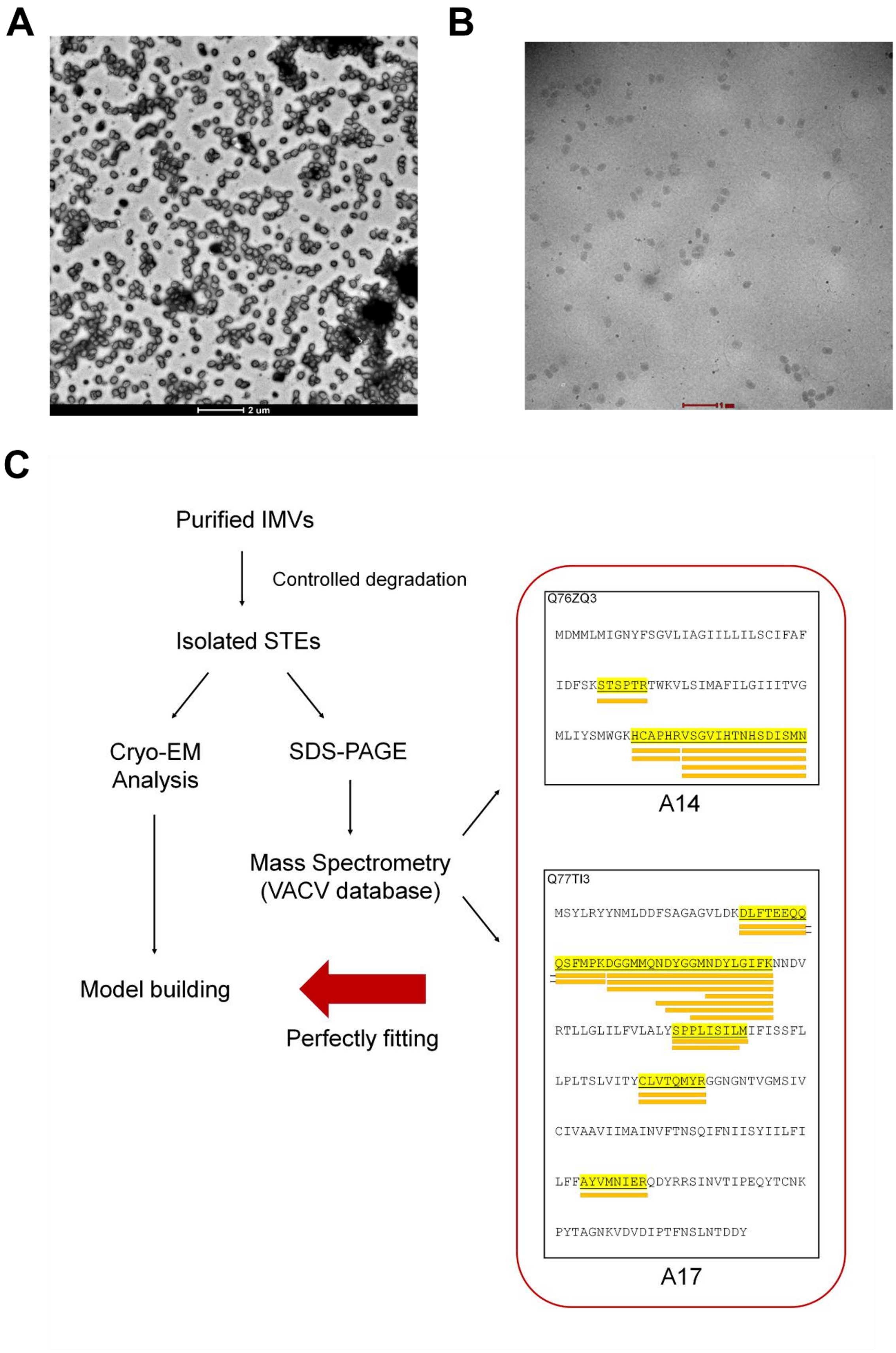
A
2 um
B
C
Purified IMVs
Controlled degradation
Isolated STEs
Cryo-EM Analysis
SDS-PAGE
Mass Spectrometry (VACV database)
Model building
Perfectly fitting
Q76ZQ3
MDMMLMIGNYFSGVLIAGIILLILSCIFAF
IDFSKSTSPTRTWKVLSIMAFILGIIITVG
MLIYSMWGKHCAPHRVSGVIHTNHSDISMN
A14
Q77TI3
MSYLRYYNMLDDFSAGAGVLDKDLFTEEQQ
QSFMPKDGGMMQNDYGGMNDYLGIFKNNDV
RTLLGLILFVLALYSPPLISILMIFISSFL
LPLTSLVITYCLVTQMYRGGNGNTVGMSIV
CIVAAVIIMAINVFTNSQIFNIISYIILFI
LFFAYVMNIERQDYRRSINVTIPEQYTCNK
PYTAGNKVDVDIPTFNSLNTDDY
A17

Fig S1 Purification and protein component determination of STEs. (A and B) MV images acquired via negative staining electron microscopy and 200 kV electron microscopy, respectively. **(C)** Following the purification of IMVs, STEs were separated using controlled degradation. Proteins of different molecular weights were resolved via SDS-PAGE, and high-coverage identification of A14 and A17 was performed using mass spectrometry. Cryo-EM analysis of STEs revealed that the atomic models of A14 and A17 were perfectly fitted into the electron density maps, confirming that A14 and A17 are the protein components of STEs.

Supplementary Figure 2

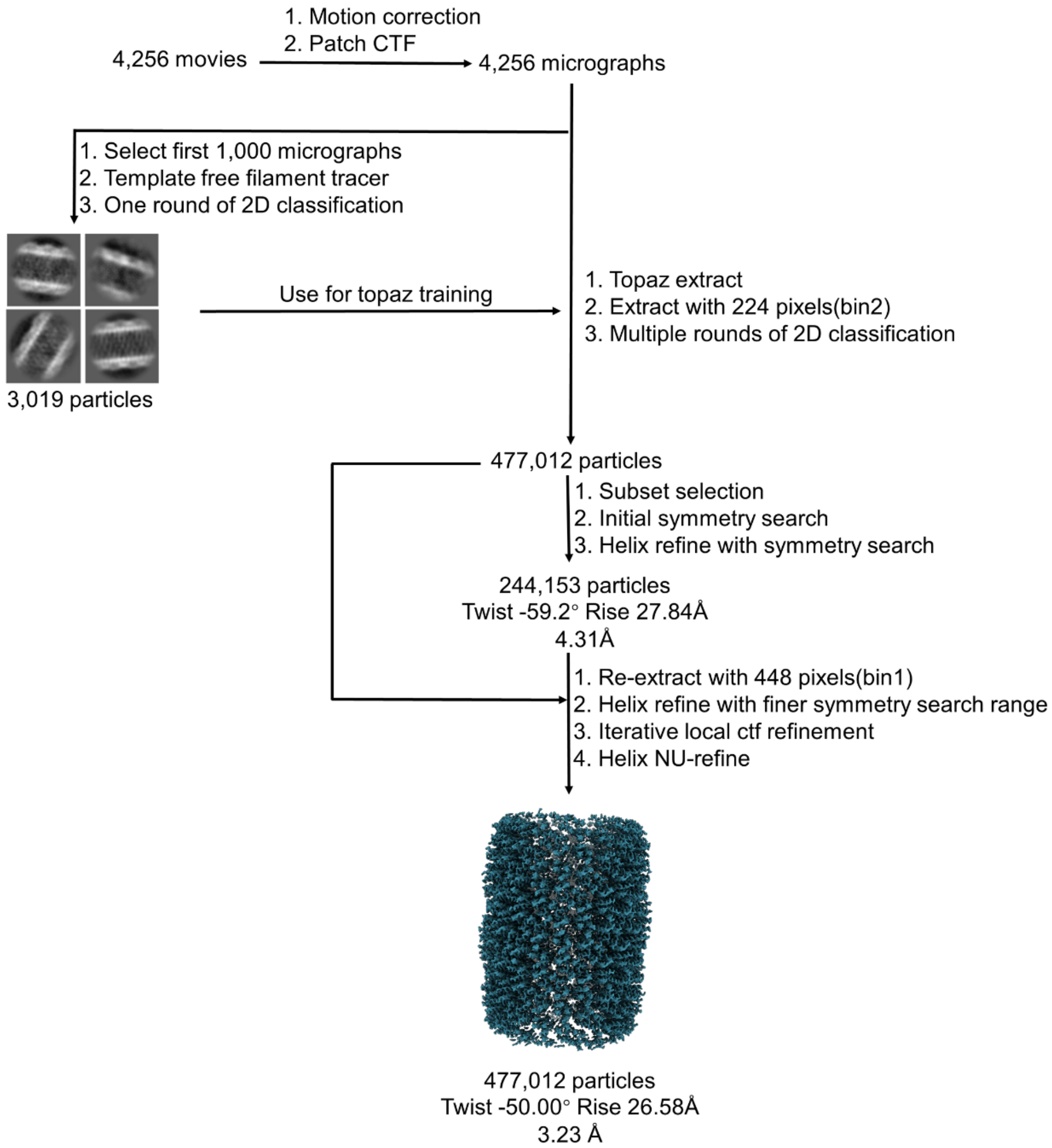

Fig S2 Helical reconstruction pipeline for VACV STE. Overview of cryo-EM image processing pipeline in cryoSPARC[1]. See Material and Methods for details.

Supplementary Figure 3

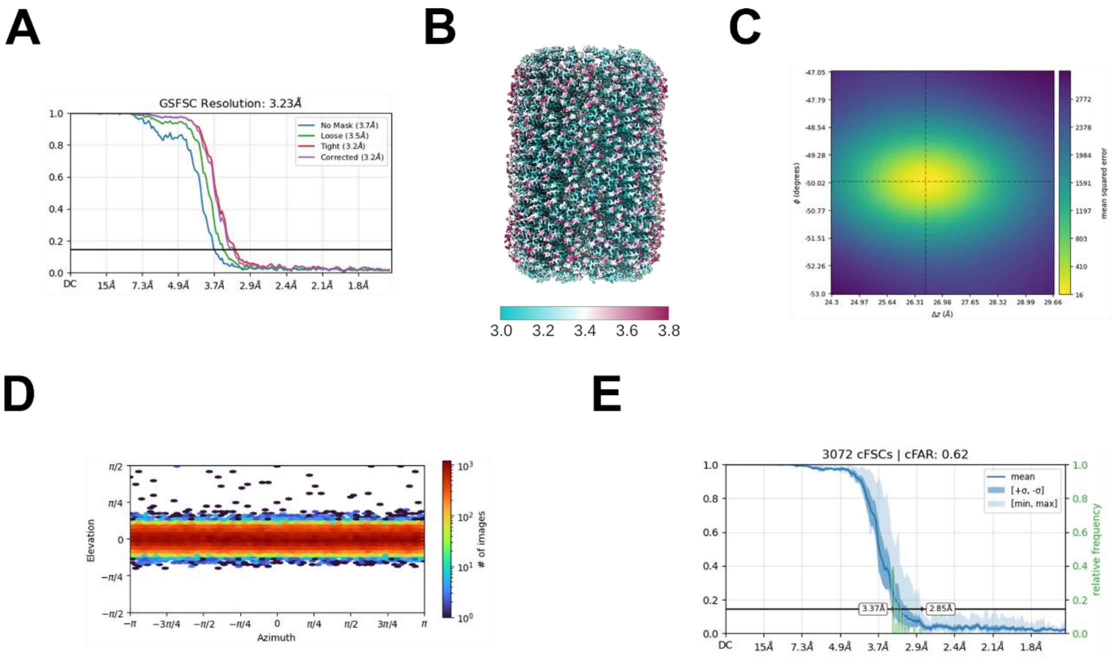

Fig S3 FSC, Local resolution and Parameter Refinement. (A) Fourier shell correlation of density map with the reported resolutions at FSC = 0.143 threshold. **(B)** Local resolution estimation of density map. **(C)** Helical symmetry error surface of density map. **(D)** Angular distribution heatmap of particles used for the refinement. **(E)** cFSCs of helical density map.

Supplementary Figure 4

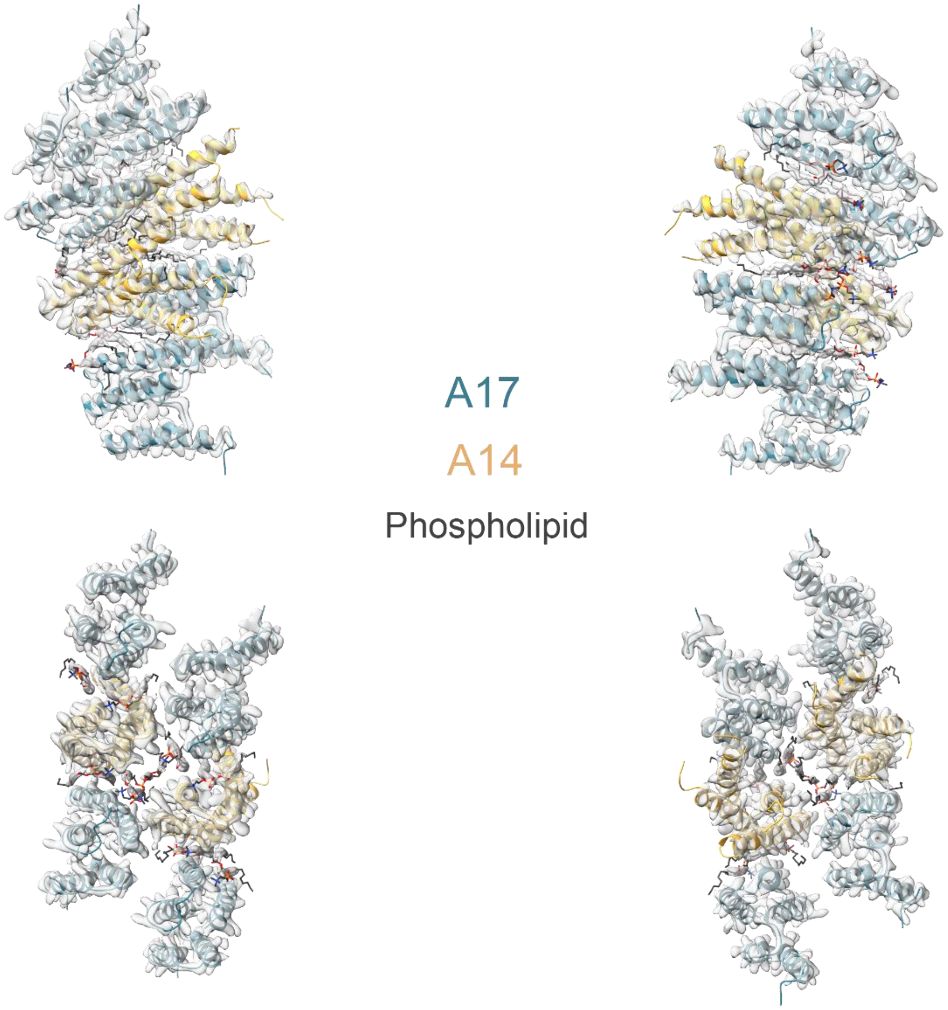

Fig S4 Molecule model of asymmetric units fit into the cryoEM density map.

Supplementary Figure 5

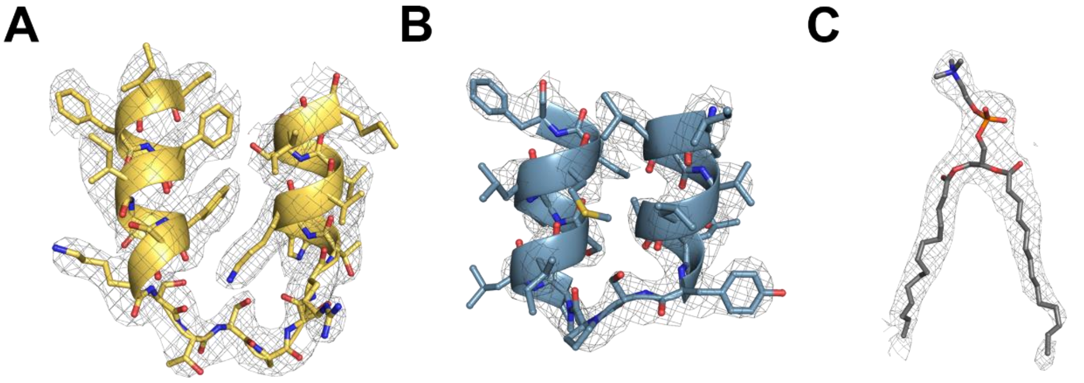

Fig S5 CryoEM density maps (mesh) from three kinds of subunits. (A) A14 (C26-M49). **(B)** A17 (I67-F85). **(C)** Phospholipid molecule.

Supplementary Figure 6

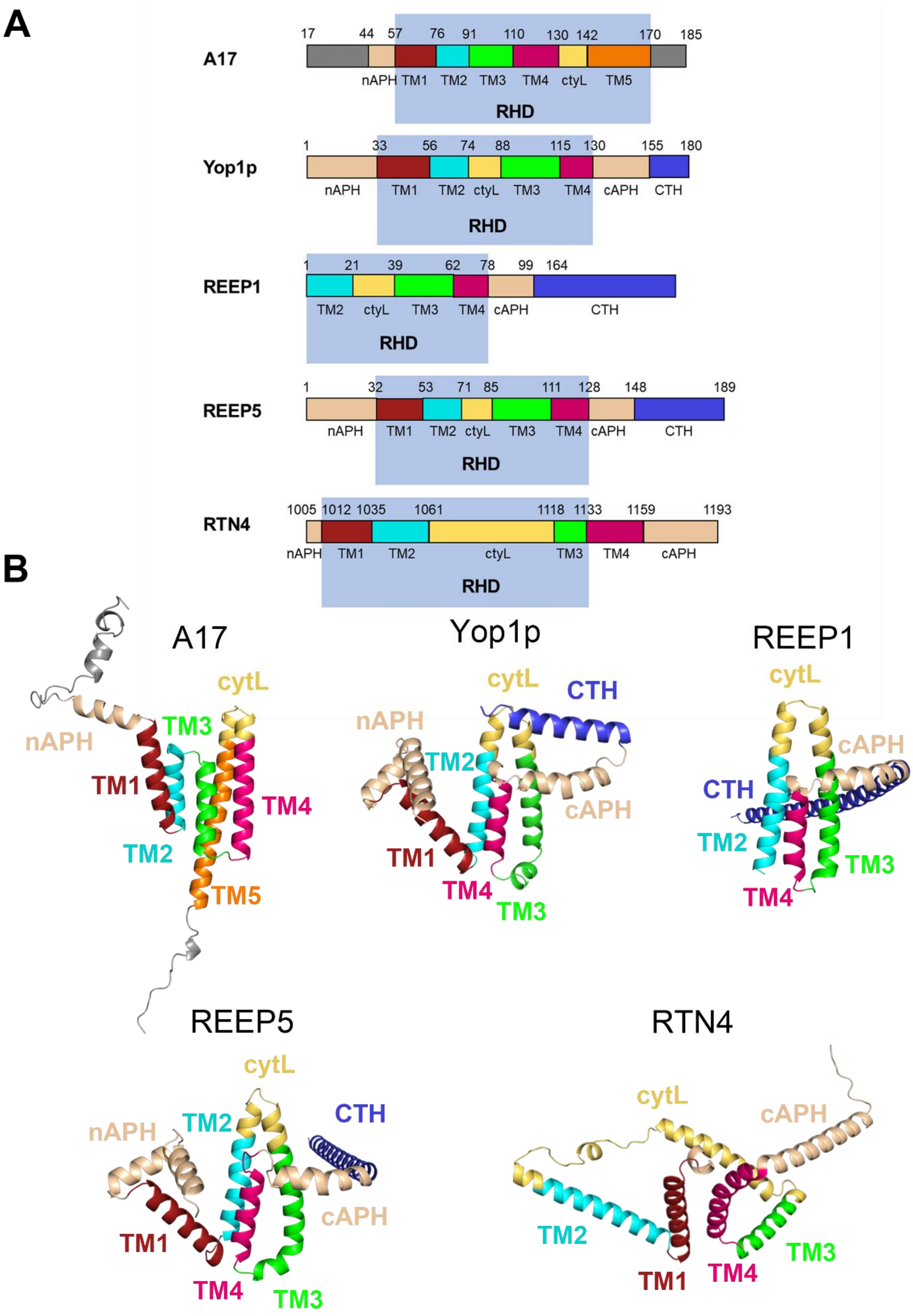
A
A17
17 44 57 76 91 110 130 142 170 185
nAPH TM1 TM2 TM3 TM4 ctyL TM5
RHD
Yop1p
1 33 56 74 88 115 130 155 180
nAPH TM1 TM2 ctyL TM3 TM4 cAPH CTH
RHD
REEP1
1 21 39 62 78 99 164
TM2 ctyL TM3 TM4 cAPH CTH
RHD
REEP5
1 32 53 71 85 111 128 148 189
nAPH TM1 TM2 ctyL TM3 TM4 cAPH CTH
RHD
RTN4
1005 1012 1035 1061 1118 1133 1159 1193
nAPH TM1 TM2 ctyL TM3 TM4 cAPH
RHD
B
A17
cytL
TM3
nAPH
TM1
TM4
TM2
TM5
Yop1p
cytL
CTH
nAPH
TM2
cAPH
TM1
TM4
TM3
REEP1
cytL
cAPH
CTH
TM2
TM3
TM4
REEP5
cytL
CTH
nAPH
TM2
cAPH
TM1
TM4
TM3
RTN4
cytL
cAPH
TM2
TM3
TM1
TM4

Fig S6 Predicted structure comparison of A17 and other ER tubule-forming proteins. (A) Schematic diagram of A17 (Vaccinia Virus), Yop1p (*Saccharomyces cerevisiae*), REEP1 (Human, residues 1-164), REEP5 (Human), and RTN4 (Human, residues 1005-1192). Key structural features are highlighted in distinct colors, including transmembrane domains (TM1-TM5), the APH (amphipathic helices), cytL (cytoplasmic loop), and CTH (C-terminal helices). Reticulon homology domains (RHD) are specifically labeled. **(B)** Structural comparison of AlphaFold3 (AF3)-predicted models of above proteins.

Supplementary Figure 7

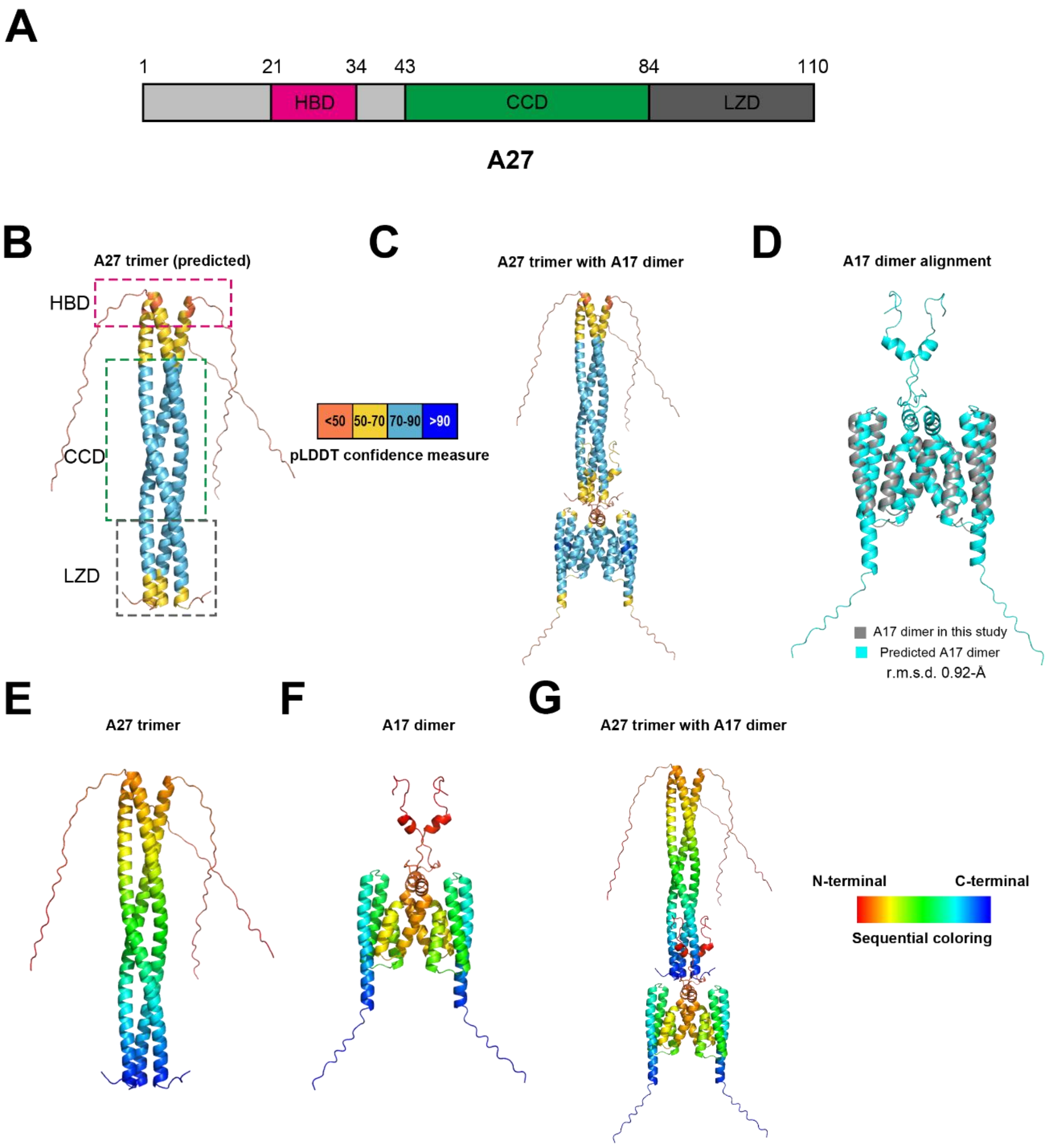

Fig S7 Predicted structures of A27 and A27-A17 complex using AlphaFold3. (A) The structural layout of the A27 sequences, highlighting the heparin-binding domain (HBD), the coiled-coil domain (CCD) for trimer assembly, and the leucine zipper domain (LZD) for binding

with A17. **(B and C)** Alphafold3 predicted structures of full-length A27 trimer and A27 trimer-A17 dimer complex, with residues colored according to the predicted local difference test (pLDDT) confidence. **(D)** Alignment between of predicted structure of A17 dimer (cyan) and the experimentally determined A17 dimer structure (color in gray). **(E–G)** Alphafold3 predicted structures of A27 trimer, A17 dimer, and A27 trimer-A17 dimer complex, with a rainbow-colored gradient from the N-terminus (red) to the C-terminus (blue).

Supplementary Figure 8

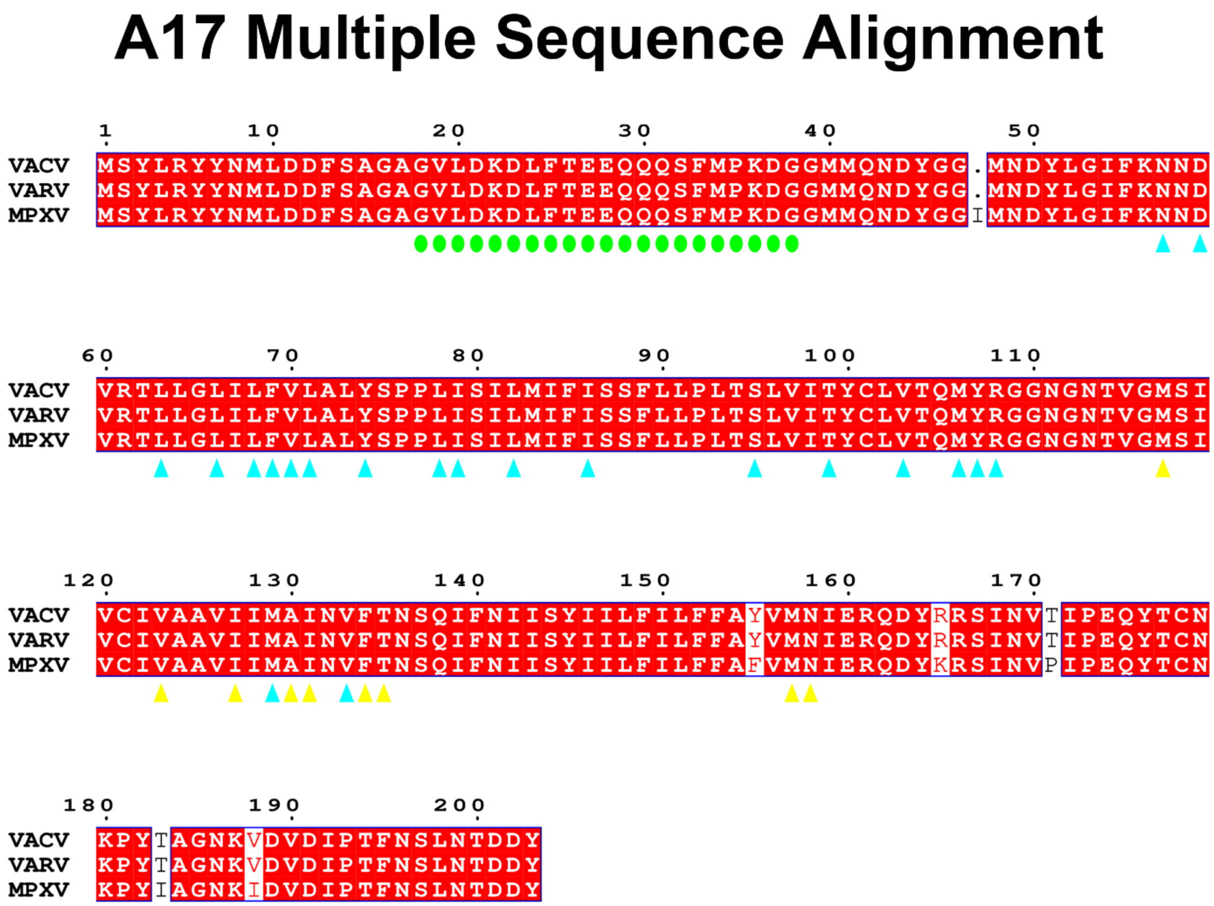

Fig S8 Sequence alignment of A17 in *Poxviridae*. The yellow and cyan triangles mark residues interacting with A14 and A17 respectively. The green circles mark the residues interacting between A17 and A27 in the prediction structure. Full names for the abbreviations: VACV, Vaccinia virus; VARV, Variola virus; MPXV, Monkeypox virus; CMLV, Camelpox virus; CPXV, Cowpox virus; HSPV, Horsepox virus; FWPV, Fowlpox virus; ORFV, Orf virus. Generated with ENDscript server [2].

Supplementary Figure 9

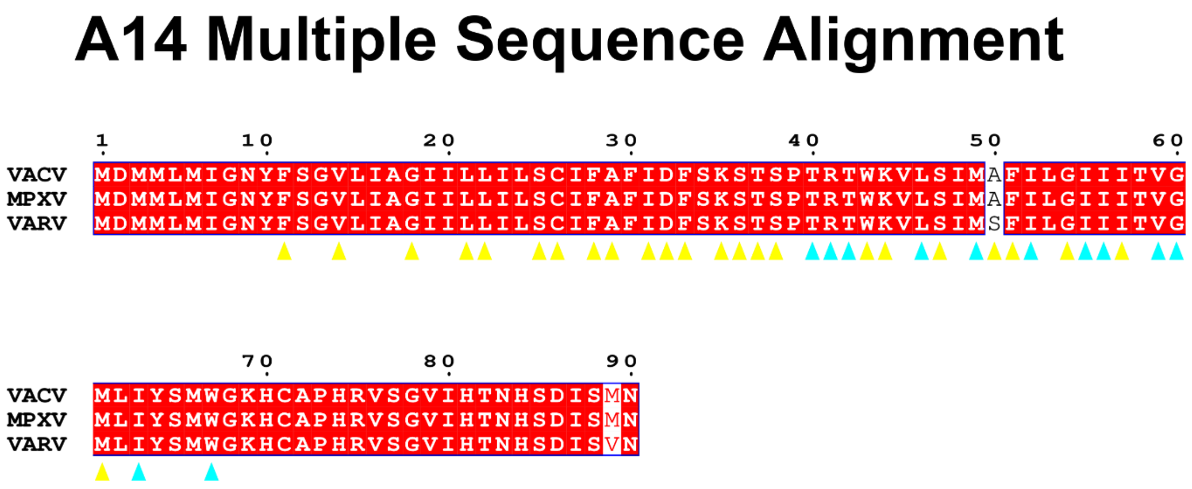

Fig S9 Sequence alignment of A14 in *Poxviridae*. The yellow and cyan triangles mark residues interacting with A14 and A17 respectively. Full names for the abbreviations are same as in Figure S8. Generated with ENDscript server [2].

Supplementary Figure 10

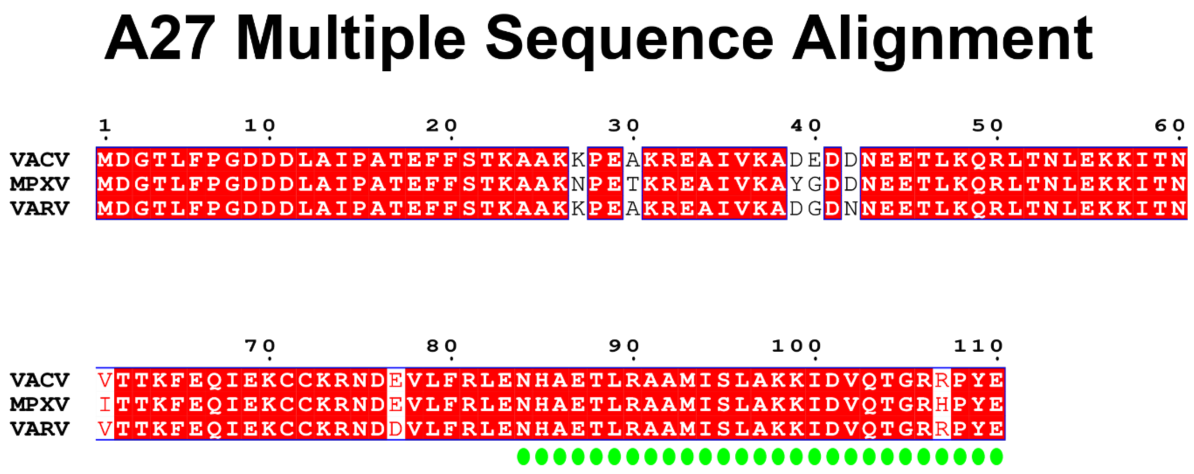

Fig S10 Sequence alignment of A27 in *Poxviridae*. The green circles mark the residues interacting between A17 and A27 in the prediction structure. Full names for the abbreviations are same as in Figure S8. Generated with ENDscript server [2].

Supplementary Tables

Table S1 Cryo-EM data collection, refinement and validation statistics

VACV Surface Tubular Element	
**Data Collection**	
Magnification	29,000
Voltage (kV)	300
Electron exposure (e⁻/Å²)	60
Defocus range (μm)	-2.5 to -1.0
Pixel size (Å)	0.82
Number of movies	4,256
**Helical Reconstruction**	
Initial particle images (no.)	1,191,313
Final particle images (no.)	477,012
Symmetry imposed	C1 followed by helical symmetry
Helical twist (˚)	-50.00
Helical rise (Å)	26.58
Map resolution (Å)<br>FSC threshold	3.23<br>0.143
**Refinement**	**VACV STE (asymmetric unit per helical turn)**
Initial model used	de novo
(PDB)	
Model resolution (Å)<br>FSC threshold	3.23<br>0.143
Map sharpening *B* factor (Å²)	-136.5
Model composition<br>Non-hydrogen atoms<br>Protein residues<br>Ligands (Phospholipid)	 5586<br>664<br>9
B factors (Å²)<br>Protein<br>Ligand	 77.35<br>83.65
R.m.s. deviations<br>Bond lengths (Å)<br>Bond angles (°)	 0.005<br>0.937
Validation<br>MolProbity score<br>Clashscore<br>Rotamers outliers (%)	 1.79<br>7.81<br>0.31
Ramachandran plot<br>Favored (%)<br>Allowed (%)	 94.75<br>4.94

Outliers (%)	0.31

Table S2 Interactions between A14 dimer

First A14 Residues	Contacts[a]	Second A14 Residues
Phe11	1	Met61
Val14	1, 2	Ile57, Met61
Gly18	1	Gly54
Leu21	2	Ala50
Leu22	1, 1	Cys26, Phe51
Ser25	1, 6, 2	Cys26 (1)[b], Ser47 (2), Ala50, Phe51
Cys26	1, 1, 1	Leu22, Ser25 (1), Cys26 (1)
Phe28	3, 2	Trp43, Ser47
Ala29	1	Ala29
Ile31	2	Trp43
Asp32	3, 2, 1, 2	Phe33, Ser38, Trp43, Lys44
Phe33	2, 4	Ala29, Asp32
Lys35	1, 1	Pro39, Trp43
Ser36	4	Ser36
Thr37	4	Thr37 (1)
Ser38	1, 3	Asp32, Ser36
Trp43	3, 2, 3, 1	Phe28, Ile31, Asp32, Lys35
Lys44	1	Asp32
Ser47	3, 1	Ser25 (1), Phe28
Ala50	3, 1	Leu21, Ser25
Phe51	1, 3	Leu22, Ser25
Ile57	1, 1	Val14, Leu21
Met61	2	Val14

Corresponding to Figures 3a-d. [a]Numbers represent the number of atom-to-atom contacts between the A14 dimer, analyzed by the Contact program in the CCP4 suite[3] (with a distance cutoff of 4.0 Å). [b]Numbers in the parentheses represent the number of potential hydrogen bonds between the two residues.

Table S3 Interactions between A17-A17.

A17 A Residues	Contacts[a]	A17 B Residues
Asn57	1	Val133
Asp59	1	Pro92
Leu63	3, 1	Ser95, Met129
Leu66	1	Leu66
Phe69	2, 1	Leu66, Val70
Val70	1, 1	Thr99, Val103
Tyr74	1, 2, 3	Tyr74 (1)[b], Val103, Arg108 (1)
Ser95	3	Leu63
Thr99	1	Val70
Val103	1, 1	Val70, Tyr74
Tyr107	3	Leu71, Tyr74
Arg108	2	Tyr74
**A17 A Residues**	**Contacts**	**A17 C Residues**
Leu68	1	Ile86
Leu71	1	Leu82
Ile86	1	Leu68
**A17 A Residues**	**Contacts**	**A17 D Residues**
Met106	1, 1	Leu78, Ile79
Tyr107	2	Ile79

Corresponding to Figures 3e-h. [a]Numbers represent the number of atom-to-atom contacts between A17 and A17, analyzed by the Contact program in the CCP4 suite[3] (with a distance cutoff of 4.0 Å). [b]Numbers in the parentheses represent the number of potential hydrogen bonds between the two residues.

Table S4 Interactions between A14-A17.

A14 E Residues	Contacts [a]	A17 F Residues
Thr40	3	Asn158(2) [b]
Arg41	2, 1	Met157(1), Asn158
Thr42	1, 1, 2	Met117, Ala154, Asn158(1)
Leu46	1	Ile150
Met49	5	Phe153
**A14 E Residues**	**Contacts**	**A17 G Residues**
Ile52	2	Val123
Ile55	1	Ile127
Ile56	1	Ala130
Val59	1	Ile131
Gly60	1	Phe134
Ile63	1	Thr135
Trp67	1	Thr135

Corresponding to Figures 3i-k. [a]Numbers represent the number of atom-to-atom contacts between A17 and A17, analyzed by the Contact program in the CCP4 suite[3] (with a distance cutoff of 4.0 Å). [b]Numbers in the parentheses represent the number of potential hydrogen bonds between the two residues.

Reference

1 Punjani, A., Rubinstein, J. L., Fleet, D. J. & Brubaker, M. A. cryoSPARC: algorithms for rapid unsupervised cryo-EM structure determination. *Nature methods* **14**, 290-296, doi:10.1038/nmeth.4169 (2017).

2 Robert, X. & Gouet, P. Deciphering key features in protein structures with the new ENDscript server. *Nucleic acids research* **42**, W320-324, doi:10.1093/nar/gku316 (2014).

3 Winn, M. D. *et al.* Overview of the CCP4 suite and current developments. *Acta crystallographica. Section D, Biological crystallography* **67**, 235-242, doi:10.1107/s0907444910045749 (2011).
